# “Turn-on” fluorescence sensor for Sc^3+^ based on Schiff-based tetraphenylethylene

**DOI:** 10.1039/d6ra06208c

**Published:** 2026-08-24

**Authors:** Shengjie Jiang, Yonglin He, Weimin Yang

**Affiliations:** a School of Petroleum and Chemical Engineering, Zhangzhou Institute of Technology ZhangZhou 363000 P. R. China jiangshengjie@fjzzit.edu.cn

## Abstract

“Turn-on” fluorescent sensors have garnered significant attention for their high signal-to-noise ratio. However, the development of aggregation-induced emission (AIE) probes for Sc^3+^ detection remains limited, with only a few examples reported to date. In this work, we rationally designed and synthesized a novel tetraphenylethylene (TPE) derivative functionalized with Schiff-base-bridged isophthalhydrazide, achieving a satisfactory yield of 85%. The probe exhibits prominent AIE characteristics and demonstrates exceptional selectivity toward Sc^3+^ over a wide range of competing metal ions, including representative lanthanides, in THF/H_2_O (5 : 95, v/v) mixed medium, accompanied by a significant fluorescence “turn-on” response upon Sc^3+^ binding. The detection limit was determined to be 3.57 × 10^−8^ M. A comprehensive mechanistic investigation, including fluorescence titration, FT-IR, ^1^H NMR, and mass spectrometry, unequivocally established a 1 : 1 binding stoichiometry between the probe and Sc^3+^. Furthermore, the practical applicability of this sensor was successfully validated through test strip experiments and simulated water sample analyses, highlighting its potential for environmental monitoring and Sc^3+^ detection in aqueous-organic mixed media.

## Abstract 2

“Turn-on” fluorescent sensors have garnered significant attention for their high signal-to-noise ratio.

## Graphical abstract

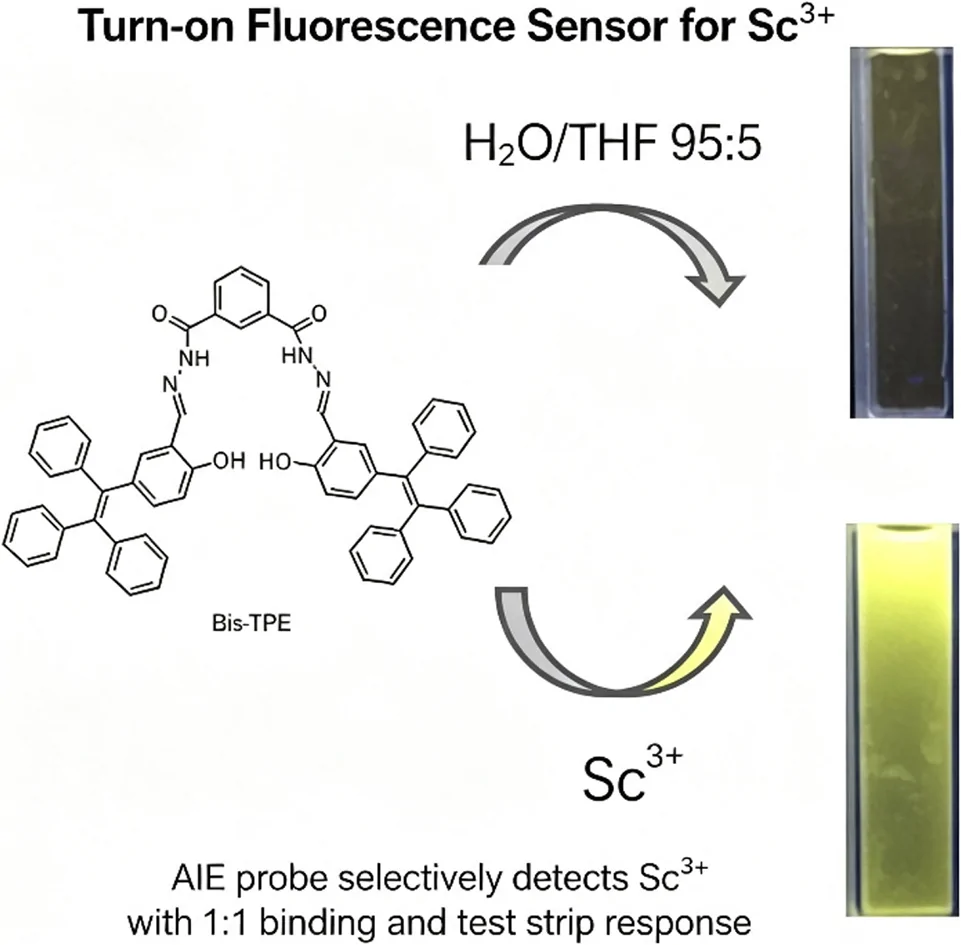

## Introduction

Scandium, as one of the rare earth elements, has found increasingly widespread applications in high-tech fields in recent years owing to its unique physicochemical properties, such as aerospace alloys, solid oxide fuel cells, laser crystals, catalysts, and nuclear medicine.^1–3^ However, due to its extremely low concentration and its typical occurrence as a concomitant element in ores of aluminum, titanium, uranium, and others, the separation and purification processes of scandium are complex and costly.^4–6^ With the increasing demand for scandium in strategic emerging industries such as clean energy, electronic information, and biomedicine, the need for real-time, sensitive, and selective detection of scandium has become increasingly urgent.^7^

However, the widespread application of scandium is also accompanied by potential environmental risks.^8–10^ Emissions from mining, metallurgy, and industrial waste release scandium into ecosystems, and its accumulation in soil and water may have toxic effects on organisms, such as inhibiting algal growth or affecting plant element absorption. Therefore, the monitoring and quantitative analysis of trace scandium ions (Sc^3+^) in environmental and industrial samples are crucial for environmental risk assessment, resource recycling, and quality control.

Traditional scandium detection methods mainly include inductively coupled plasma mass spectrometry (ICP-MS), inductively coupled plasma optical emission spectrometry (ICP-OES), atomic absorption spectrometry (AAS), X-ray fluorescence spectrometry (XRF), and neutron activation analysis (NAA).^6,11–14^ Although these methods exhibit high sensitivity and accuracy, they are challenging to meet the needs of on-site rapid detection and real-time monitoring due to their expensive equipment, complex operation, and cumbersome sample pretreatment. Therefore, developing a simple, rapid, low-cost, and field-friendly method for scandium ion analysis has become a current research hotspot.

To overcome the aforementioned limitations, the development of a simple, rapid, low-cost Sc^3+^ analysis method suitable for on-site detection has become a research hotspot. Among them, colorimetric methods are favored due to their simple instrumentation, convenient operation, rapid response, and even direct visual interpretation.^15–18^ However, most existing colorimetric methods still suffer from deficiencies in sensitivity and selectivity, especially the difficulty in effectively eliminating interference from other rare earth ions with similar physicochemical properties. Therefore, the development of a novel Sc^3+^ colorimetric sensing strategy with high sensitivity and selectivity holds significant scientific importance and application value.^19–21^ Moreover, normal fluorescence sensors easily aggregated in the aqueous-organic mixed medium and caused the strong ACQ effect, which greatly limited the application in the actual environment and *in vivo*.^18,22–27^ In 2001, Tang's research group discovered aggregation-induced emission (AIE) which provided a new opinion for constructing novel fluorescent sensors in aqueous-organic mixed medium. In this work, based on design and synthesis of a Schiff-base bridged isophthalic dihydrazide tetraphenylethylene. Although several fluorescent probes for Sc^3+^ have been reported,^3,4,28^ their application remains limited, and the development of new probes with improved performance is still desirable. We wish to report a new “turn-on” AIE sensor for Sc^3+^ with high selective sensing ability in aqueous-organic mixed medium, which was further applied in detection of Sc^3+^.

## Results and discussion

### Systhesis

**Scheme 1:**
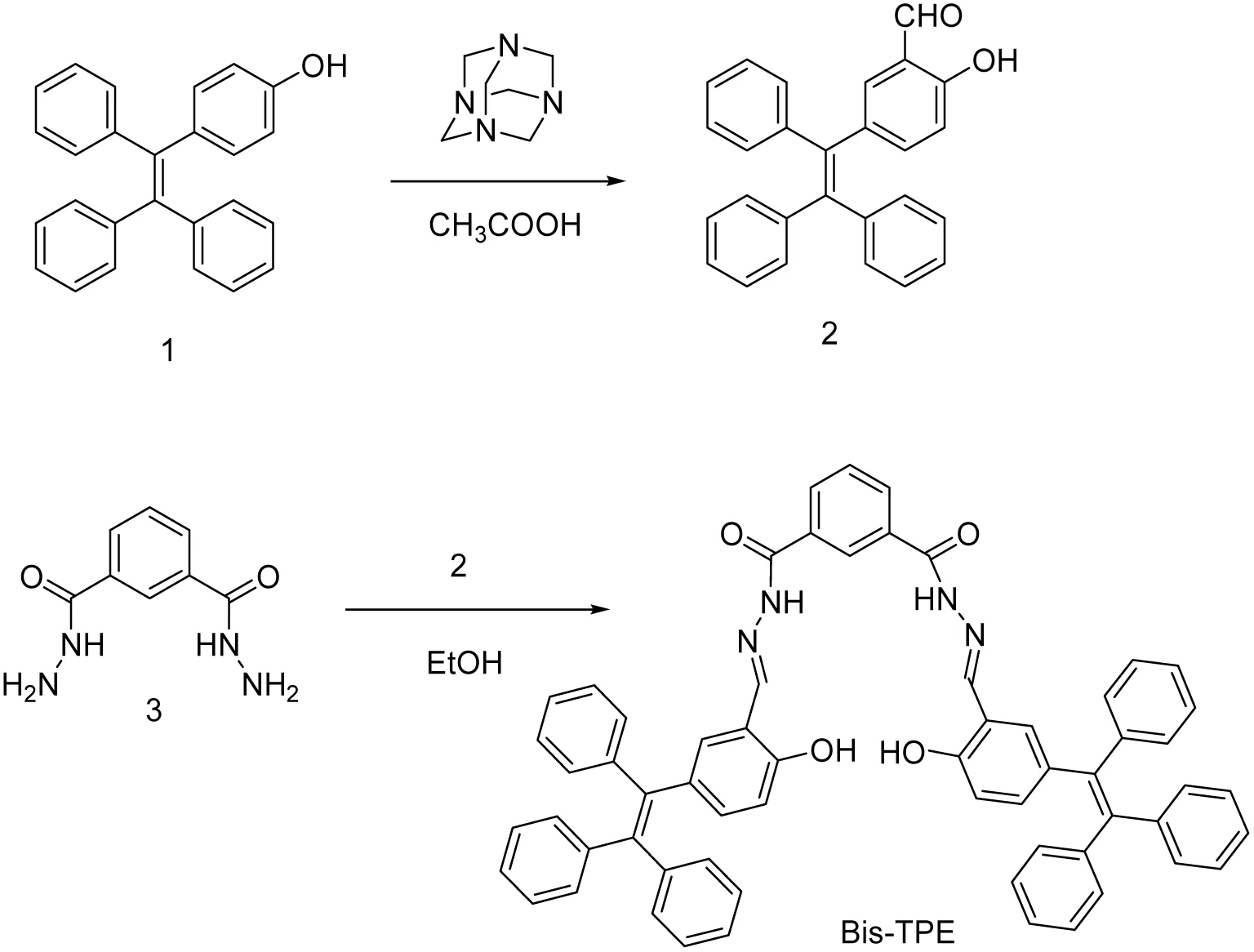
Synthesis of target acylhydrazone-bridged bis-tetraphenylethylene (Bis-TPE).

Since rare earth ions are hard acids, they are easily coordinated by coordination atoms such as hard base O, N. Therefore, the tetraphenylethylene group were bridged by Schiff base to enhance the binding ability to rare earth ions. The target compound of Schiff base bridged Bis-TPE was designed and synthesized, achieving long-wavelength luminescence. The synthesis route is shown in Scheme 1. First, compound 2 was synthesized from compound 1 in the HOAc/methenamine system in 55% yield by a classical formylation reaction according to the disclosed literature method. Finally, compound 2 and isophthalic dihydrazide were condensed by Schiff base reaction to give the target Bis-TPE, which was purified in 85% yield. The structure of Bis-TPE was fully confirmed by ^1^H NMR and ^13^C NMR spectroscopy (see SI Fig. S1 and S2). In addition, the MALDI-TOF MS spectrum is provided in Fig. S3 (SI), MALDI-TOF MS showed the expected molecular ion peaks. The elemental analysis data are consistent with the structure in Scheme 1, Bis-TPE was synthesized according to the procedure described in the SI.

### AIE property

**Fig. 1:**
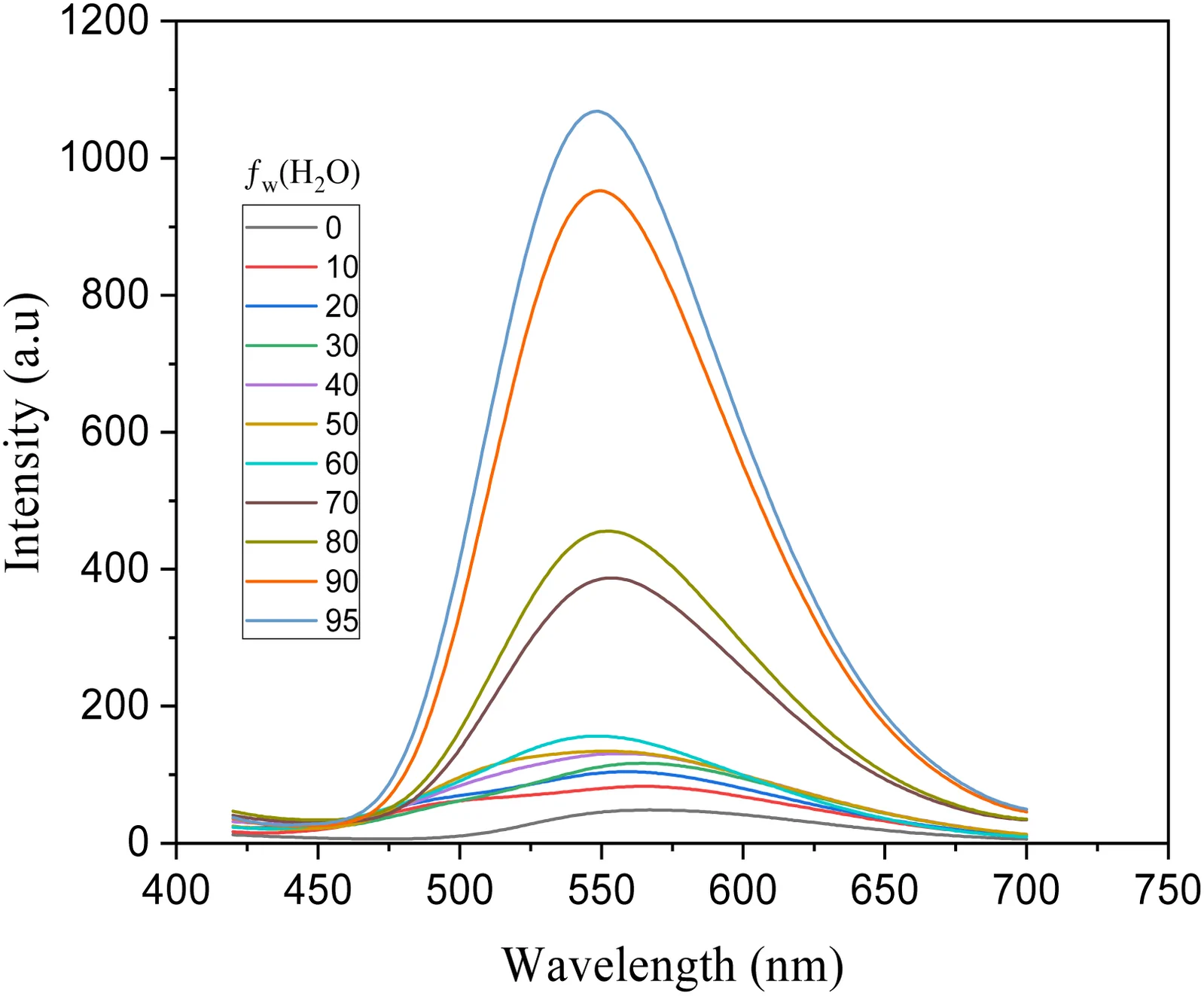
The emission spectra of Bis-TPE in THF/H_2_O solutions with different fractions of H_2_O (1 × 10^−5^ M, *λ*_ex_ = 360 nm).

**Fig. 2:**
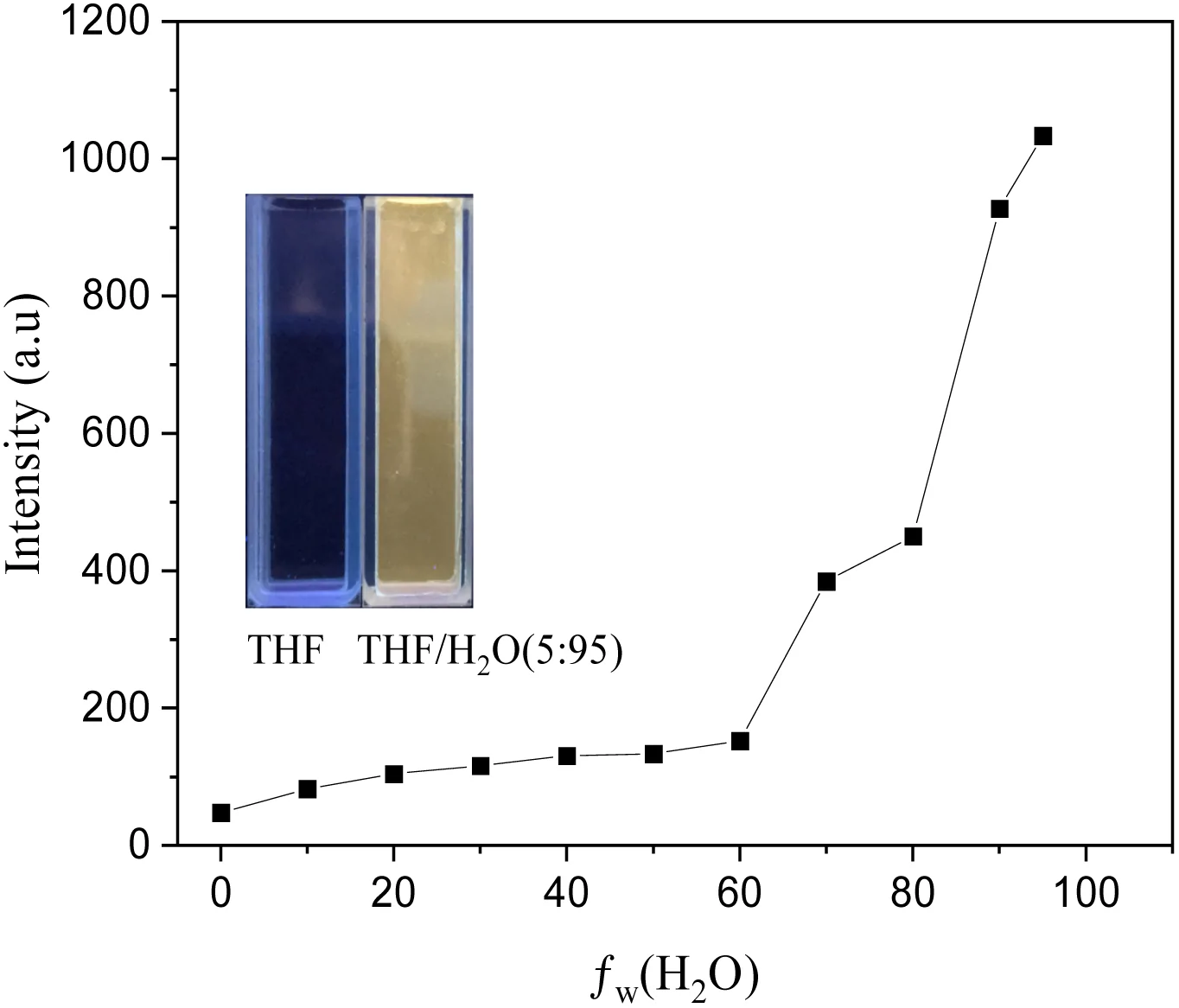
The changes of peak intensities in THF-H_2_O with different H_2_O fractions (*λ*_ex_ = 360 nm, 1.0 × 10^−5^ M).

**Fig. 3:**
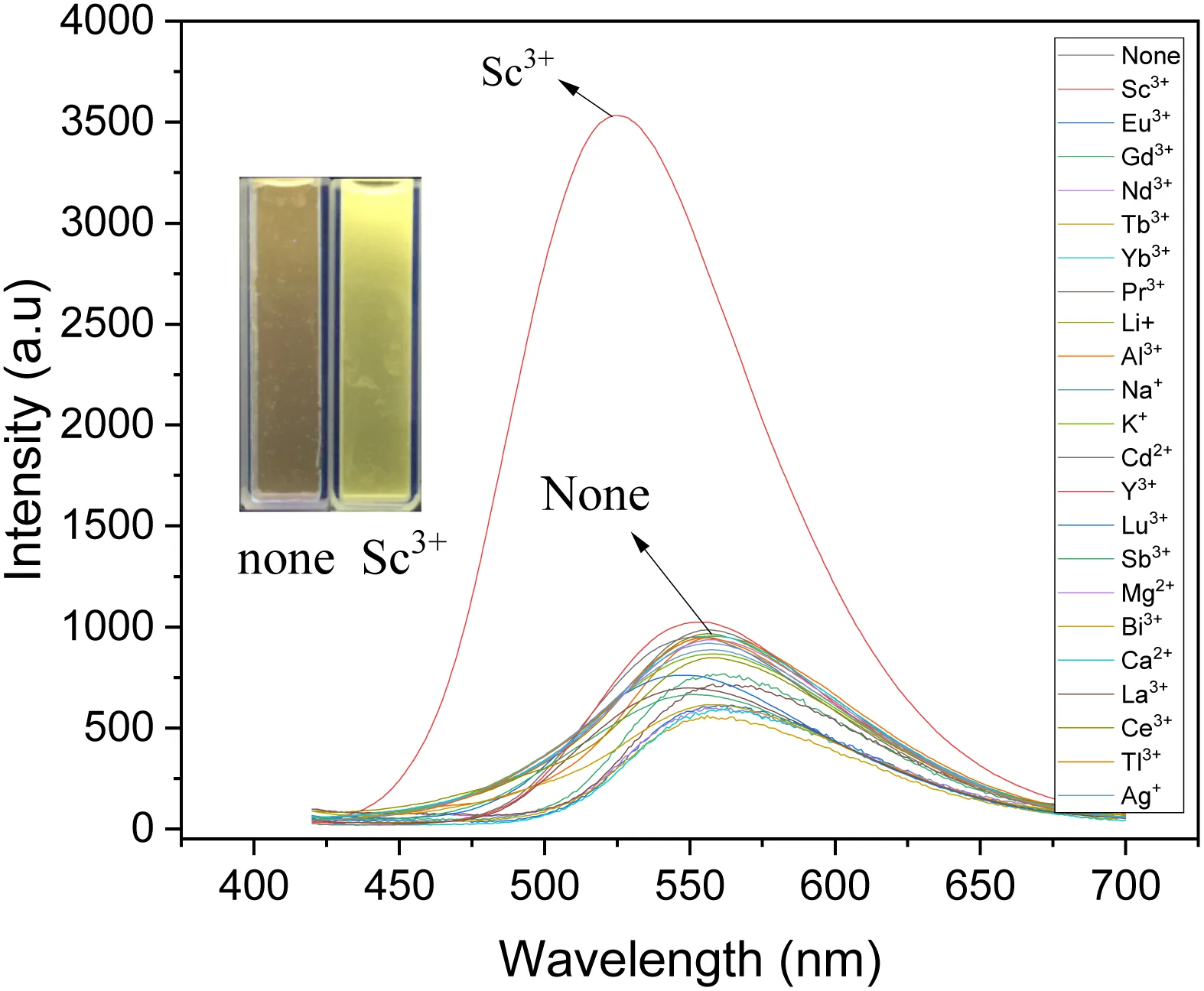
Fluorescence spectra of Bis-TPE (*λ*_ex_ = 360 nm, 1 × 10^−5^ M) with guests (1 × 10^−4^ M) in THF-H_2_O (5 : 95). (Sc^3+^, Pr^3+^, Nd^3+^, Eu^3+^, Gd^3+^, Tb^3+^, Yb^3^, Li^+^, Al^3+^, Na^+^, K^+^, Cd^2+^, Y^3+^, Lu^3+^, Sb^3+^, Mg^2+^, Bi^3+^, Ca^2+^, La^3+^, Ce^3+^, Tl^3+^, Ag^+^). Inserted images: left = Bis-TPE, right = Bis-TPE + Sc^3+^.

A series of Bis-TPE solutions with different THF/water ratios were prepared to study their AIE properties. As shown in Fig. 1, Bis-TPE exhibits good solubility in pure THF solution and very weak fluorescence emission. The significantly weak fluorescence can be explained by the active intramolecular rotation of the tetraphenylethylene group, which depletes the energy of the excited state, leading to fluorescence quenching. However, as the poor solvent increases, the fluorescence intensity sharply increases, and reaches its maximum when the water content is 95% (Fig. 2). Compared to the fluorescence intensity of Bis-TPE in pure THF solution, it is enhanced by about 9.1 times. The change in fluorescence intensity can be attributed to the aggregation state of the probe Bis-TPE as the poor solvent increases, which hinders the free rotation of the intramolecular aromatic rings and inhibits the non-radiative transition of the excited state energy. Fig. S4 hydrodynamic particle sizes of the luminogen at different water fractions measured by dynamic light scattering (DLS). The molecules are well-dissolved as isolated monomers with small and stable sizes at low water content. After the water fraction surpasses the critical point, poor-solvent-induced aggregation occurs and the particle size increases sharply, further verifying the aggregation process behind the AIE phenomenon. This results in the excited state molecules returning to the ground state only through radiative transition, thereby generating stronger fluorescence emission. Based on this, Bis-TPE is shown to be an AIE-active compound. Since Bis-TPE exhibits the strongest fluorescence emission in 95% water content, the THF-H_2_O (5 : 95) mixed system was selected as the sensing system for detecting metal ions in the following studies (Fig. 3).

### The sensing abilities for series metal ions

Investigating the selectivity and sensitivity of sensors is crucial. Therefore, we employed fluorescence spectroscopy to examine the detection capability of Bis-TPE for various metal ions in THF-H_2_O (5 : 95). By introducing a series of metal ions(Sc^3+^, Pr^3+^, Nd^3+^, Eu^3+^, Gd^3+^, Tb^3+^, Yb^3^, Li^+^, Al^3+^, Na^+^, K^+^, Cd^2+^, Y^3+^, Lu^3+^, Sb^3+^, Mg^2+^, Bi^3+^, Ca^2+^, La^3+^, Ce^3+^, Tl^3+^, Ag^+^) into the Bis-TPE solution, we studied the fluorescence changes in the mixed system. As evident from Fig. 3, compared to the fluorescence of Bis-TPE without added metal ions, the fluorescence intensity of Bis-TPE fluctuated within a narrow range after the introduction of various metal cations, except for Sc^3+^. Even though the maximum emission wavelength underwent a slight blue shift upon the addition of individual ions, these changes were minimal. However, upon the introduction of Sc^3+^ into the Bis-TPE solution, a notable increase in fluorescence emission was observed, with an enhancement of approximately 3.8 times, accompanied by a blue shift of 39 nm in the maximum emission wavelength (from 559 nm to 520 nm). As shown in the inset of Fig. 2, the naked eye could clearly perceive the enhancement of fluorescence and the change in color. These results undoubtedly support Bis-TPE selective sensing ability towards Sc^3+^ through the “turn-on” fluorescence phenomenon among all tested cations.

### Fluorescence titration for Sc^3+^

**Fig. 4:**
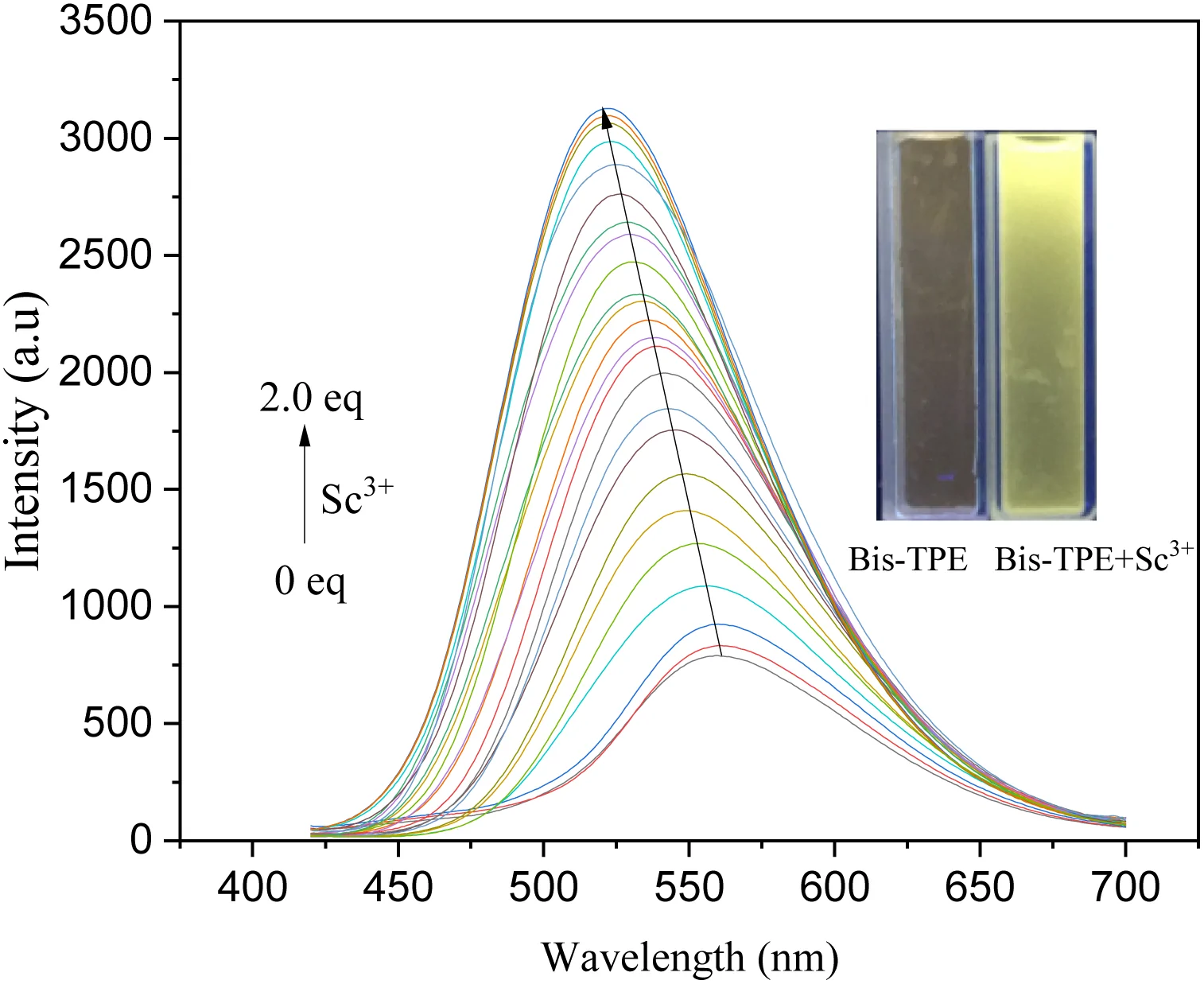
Fluorescence spectra of Bis-TPE (*λ*_ex_ = 360 nm, 5.0 × 10^−6^ M) with different equivalent concentrations of Sc^3+^ in THF-H_2_O (5 : 95). Inserted images: A = Bis-TPE, B = Bis-TPE + Sc^3+^ (0.5 eq.), C = Bis-TPE + Sc^3+^ (1.0 eq.).

**Fig. 5:**
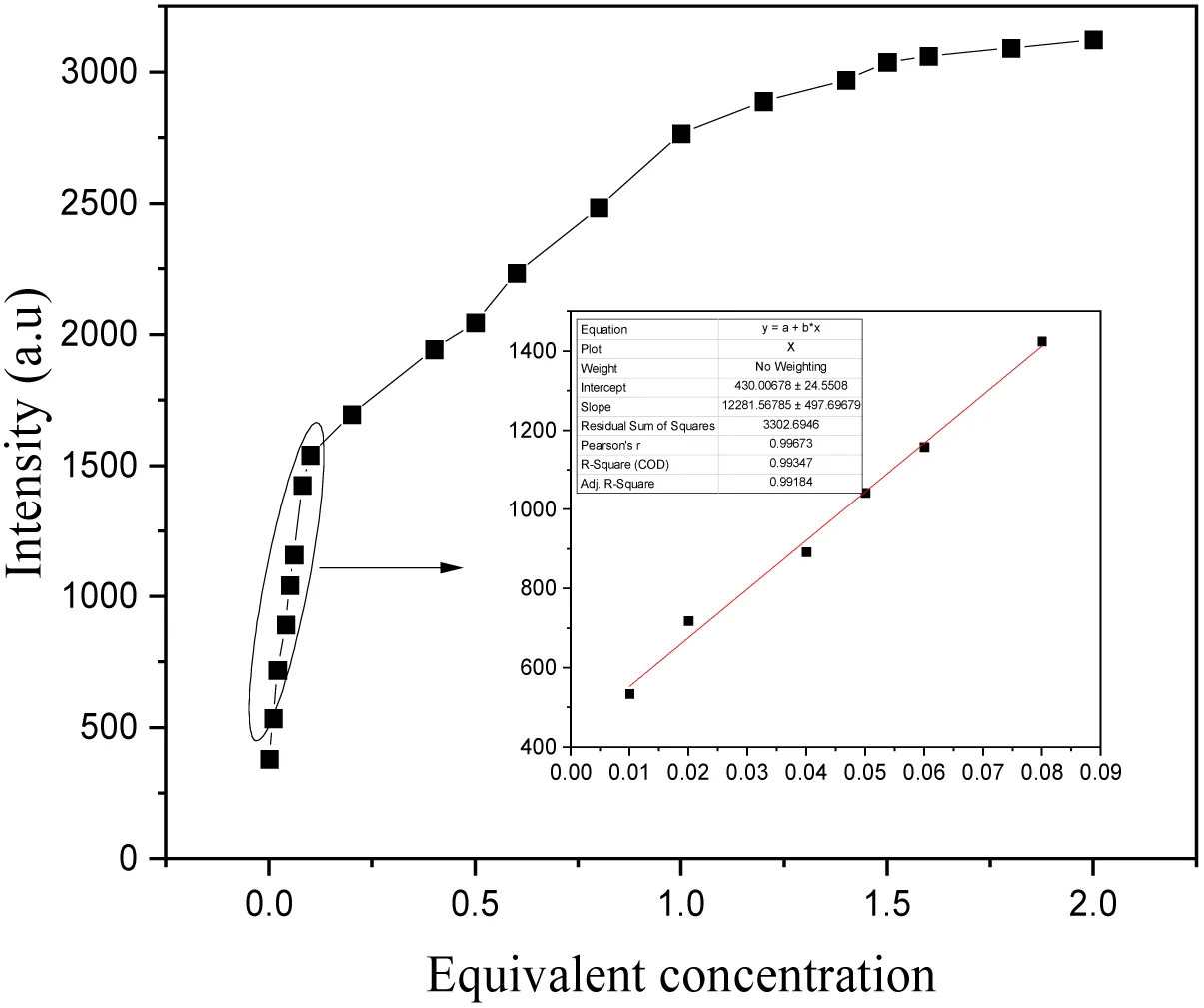
Fluorescence intensities (542 nm) of Bis-TPE with different equivalent concentrations of Sc^3+^ in THF-H_2_O (5 : 95).

The detailed sensing behavior of Bis-TPE towards Sc^3+^ was investigated through fluorescence titration in THF-H_2_O (5 : 95). As shown in Fig. 4, the fluorescence intensity gradually increases with the increase in equivalent concentration of Sc^3+^. A rapid growth is observed when the equivalent concentration of Sc^3+^ is less than 1.0, while the fluorescence intensity increases slowly when the equivalent concentration is greater than 1.0. These results indicate that the inflection point of the titration is at an equivalent concentration of 1.0, suggesting a stoichiometric ratio of Bis-TPE to Sc^3+^ of 1 : 1. Furthermore, Fig. 5 displays the corresponding curve of fluorescence intensity *versus* equivalent concentration of Sc^3+^. It can be observed that within the range of 0.0–1.0, the fluorescence intensity of Bis-TPE shows a good linear relationship with the equivalent concentration of Sc^3+^. According to the detection limit formula *D*_L_ = *K* × *S*_b1_/*S* (where *K* = 2 or 3, set to 3 here, *S*_b1_ is the standard deviation of the blank solution, and *S* is the slope value of the regression line), the detection limit of Sc^3+^ is calculated as 3.57 × 10^−8^ M. This low detection limit indicates that sensing trace amounts of Sc^3+^ has good application prospects.

### pH influence and interference experiments on sensing Sc^3+^

**Fig. 6:**
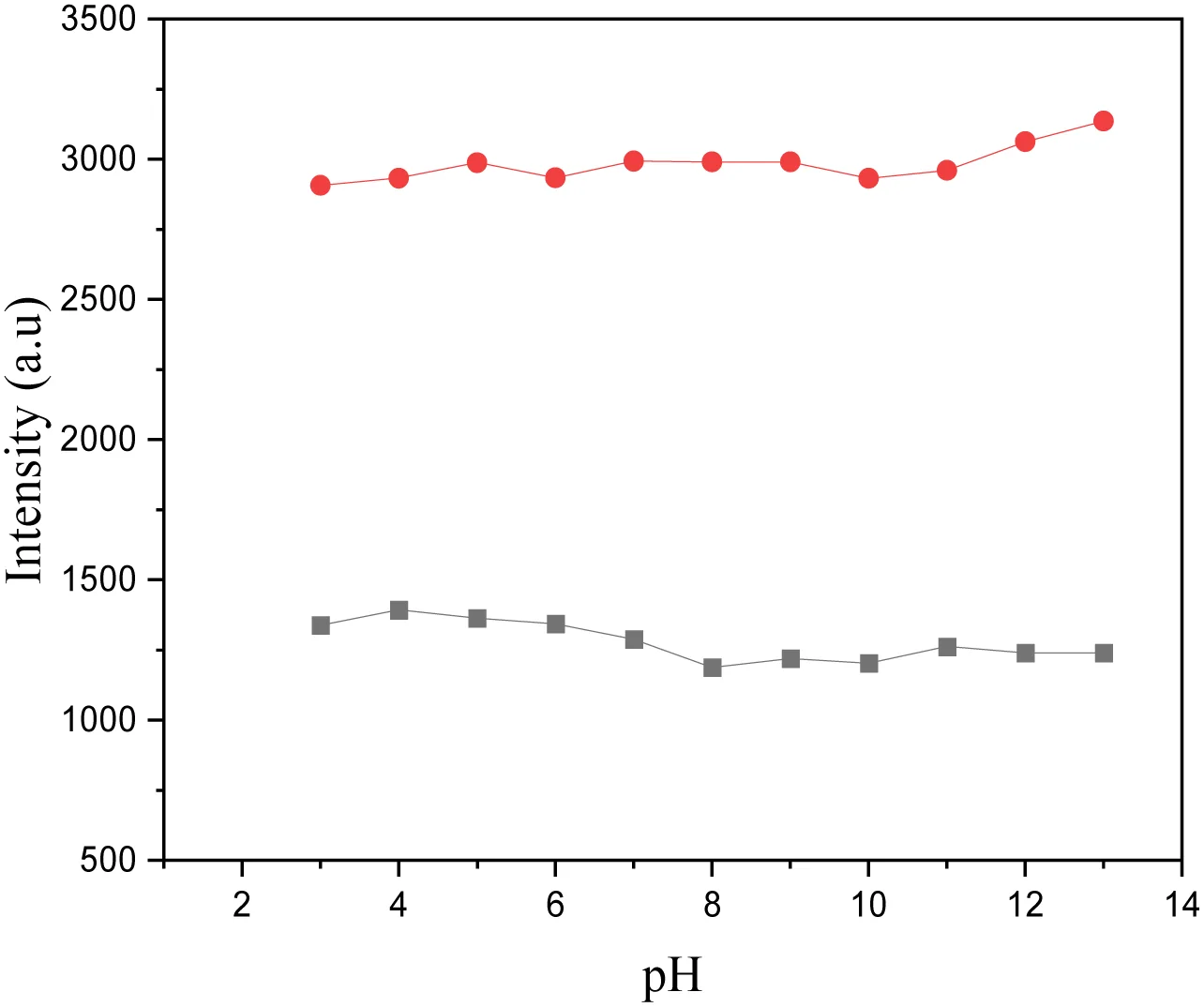
The influence of pH on the fluorescence intensities of Bis-TPE (1 × 10^−5^ M) in THF-H_2_O (5 : 95) with (red) or without (black) Sc^3+^ (1 × 10^−5^ M), *λ*_ex_ = 360 nm.

The influence of pH on the fluorescence emission of Bis-TPE in THF/H_2_O (5 : 95, v/v) in the absence and presence of Sc^3+^ was investigated, as shown in Fig. 6. The fluorescence intensity of Bis-TPE remained stable over the pH range of 2–10. Upon the addition of Sc^3+^, a significant fluorescence enhancement was observed across the same pH range, and the enhanced intensity remained stable between pH 2 and 9. However, a slight decrease was observed under strongly alkaline conditions (pH > 10), which may be attributed to the deprotonation of the phenolic hydroxyl group of the ligand to phenolate, thereby weakening the coordination ability toward Sc^3+^. These results indicate that the Bis-TPE/Sc^3+^ system exhibits stable fluorescence emission over a broad pH range (2–9), suggesting its promising potential for practical applications.

**Fig. 7:**
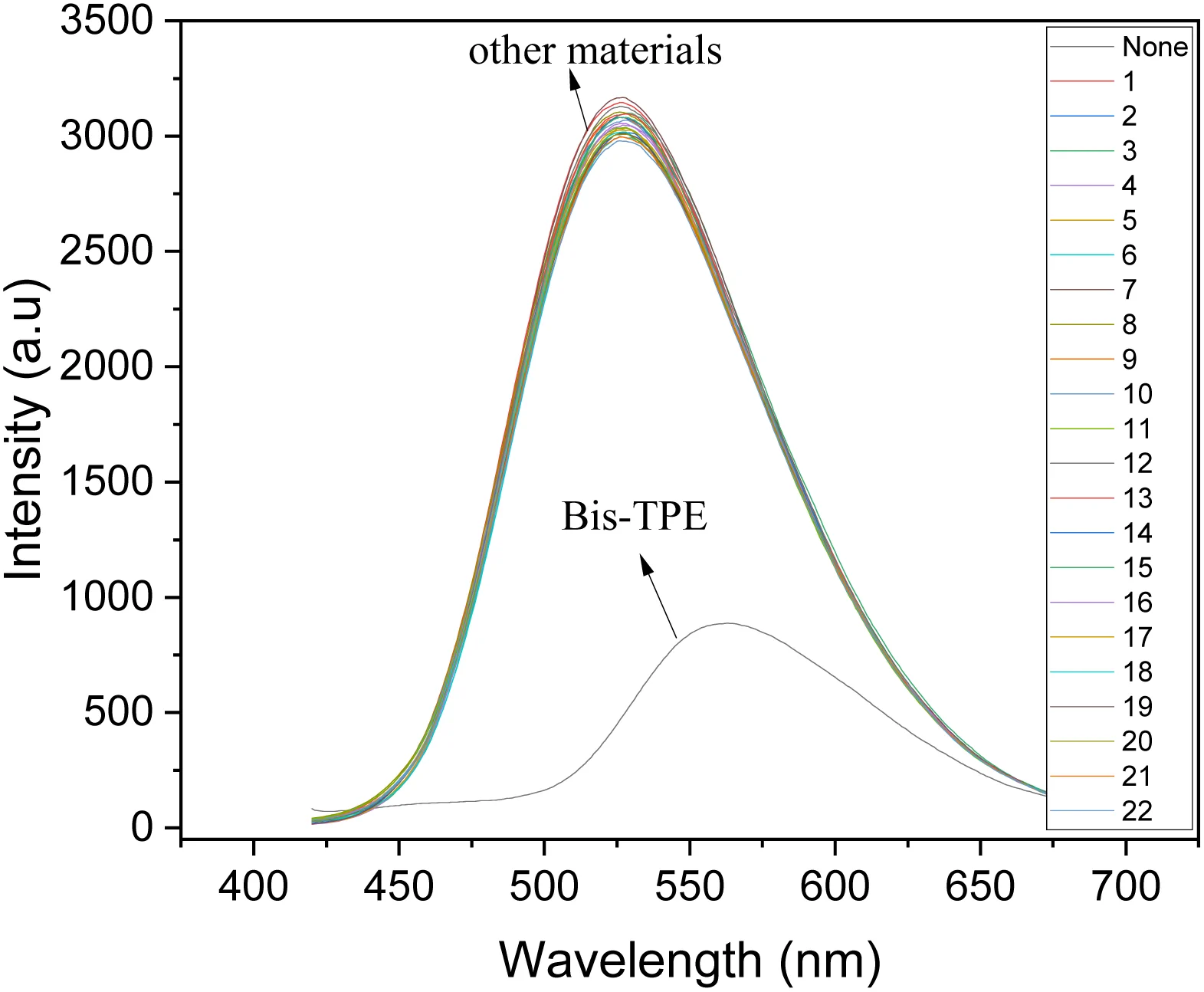
Fluorescence spectra of Bis-TPE (1 × 10^−5^ M) with Sc^3+^ and other materials (2 × 10^−5^ M each) in a mixture of THF-H_2_O (5 : 95) at *λ*_ex_ = 360 nm. (1 = Bis-TPE + Sc^3+^, 2–22 = Bis-TPE + Sc^3+^ with (Pr^3+^, Nd^3+^, Eu^3+^, Gd^3+^, Tb^3+^, Yb^3^, Li^+^, Al^3+^, Na^+^, K^+^, Cd^2+^, Y^3+^, Lu^3+^, Sb^3+^, Mg^2+^, Bi^3+^, Ca^2+^, La^3+^, Ce^3+^, Tl^3+^, Ag^+^) respectively).

The selectivity of Bis-TPE toward Sc^3+^ was further evaluated by interference experiments, as shown in Fig. 7. Free Bis-TPE exhibited weak emission at 559 nm, whereas the addition of Sc^3+^ resulted in a strong turn-on fluorescence at 520 nm with a hypsochromic shift. In the presence of various competing metal ions and organic biomolecules, the emission intensity at 520 nm remained similar to that of the Bis-TPE/Sc^3+^ system, with only minor fluctuations. These results confirm that Bis-TPE possesses high selectivity for Sc^3+^ over the tested interfering species.

### The sensing mechanism

The stoichiometric ratio of Bis-TPE to Sc^3+^ was studied through the working curve shown in Fig. S5. It can be seen that the inflection point occurs when Bis-TPE = 0.5, indicating that the stoichiometric ratio of Bis-TPE to Sc^3+^ is 1 : 1. The MS spectra of Bis-TPE with excess Sc^3+^ (5 eq.) showed a binding peak at 957.926 (Fig. S6), which was consistent with the molecular weight of (M + Sc^3+^ + H), indicating a stoichiometric ratio of 1 : 1 again.

**Fig. 8:**
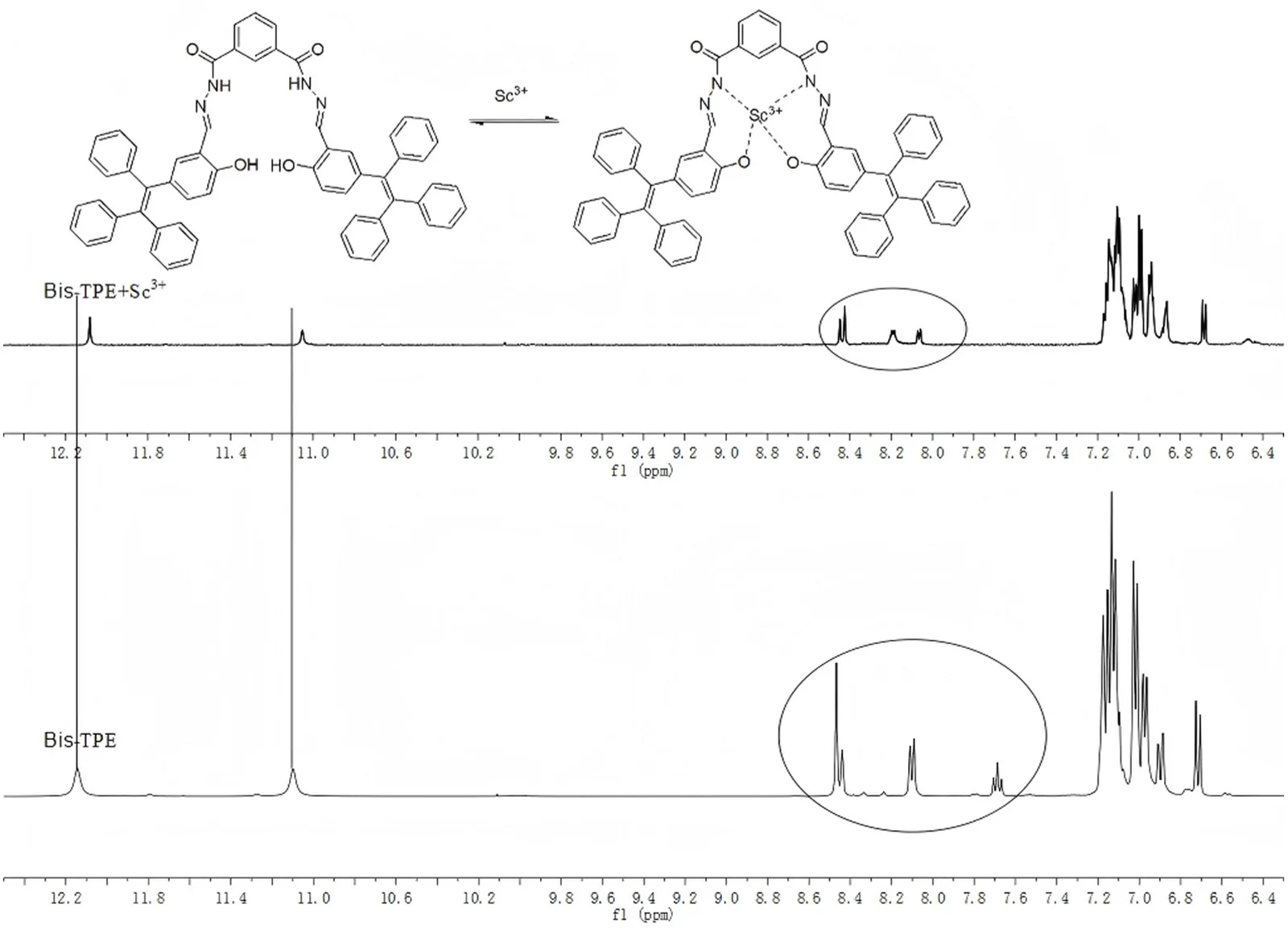
The ^1^H NMR spectrum of Bis-TPE with Sc^3+^ (1 : 1).

The sensing mechanism of Bis-TPE for Sc^3+^ was studied by IR and ^1^H NMR. As shown in Fig. S7, compared with the FT-IR spectrum of Bis-TPE, the peaks of C–O and OH functional groups in the FT-IR spectrum of Bis-TPE containing Sc^3+^ have shifted significantly, which means that carboxyl and phenolic hydroxyl groups participate in the perception of Sc^3+^. Comparison of ^1^H NMR spectra of Bis-TPE and Bis-TPE + Sc^3+^ (1 : 1) showed that the signals of OH and N=CH were offset, indicating that OH and N=CH groups were involved in the perception of Sc^3+^ (Fig. 8). Therefore, based on the above results, a possible sensing mechanism is proposed, as shown in Fig. 8.

**Fig. 9:**
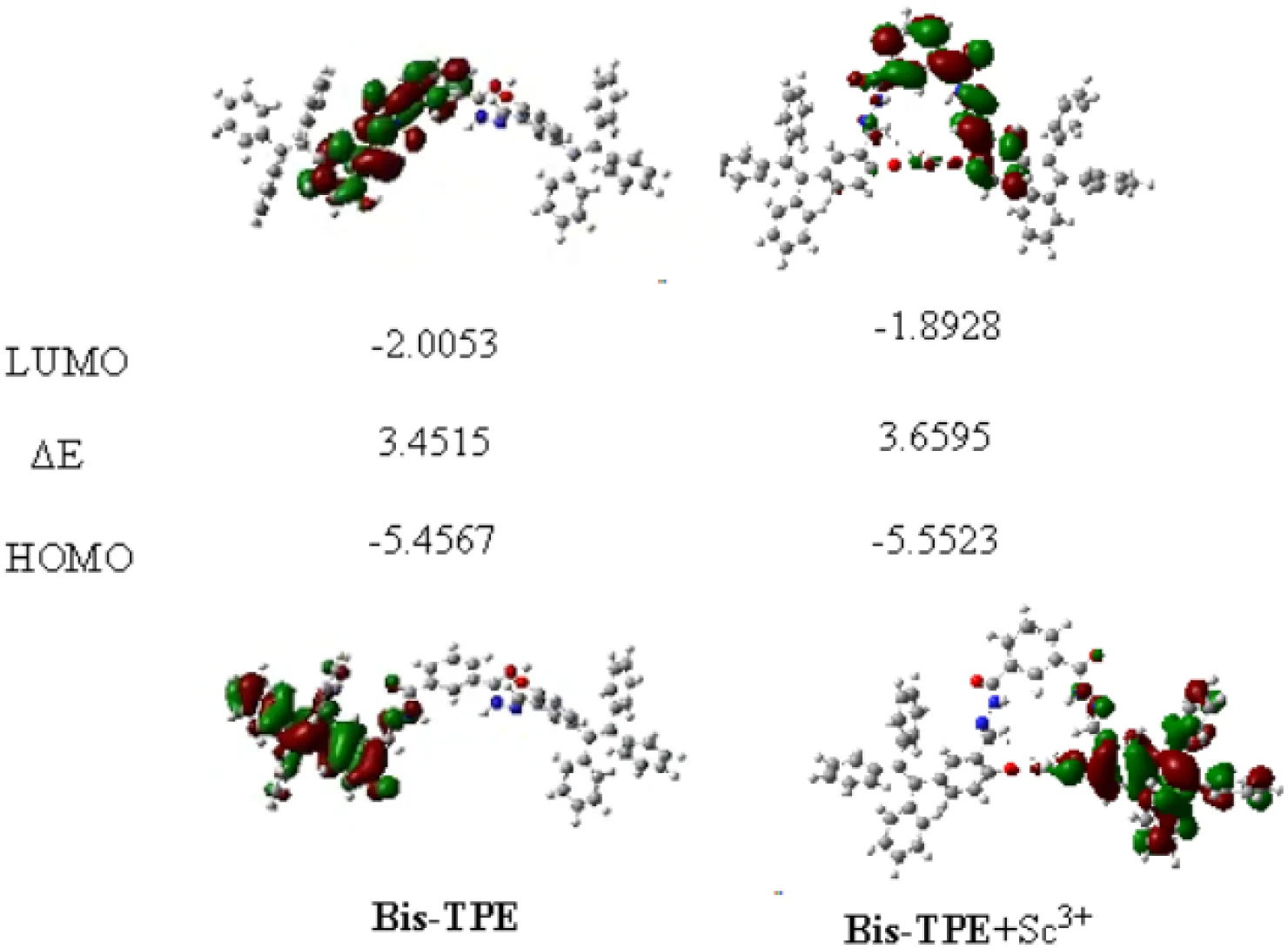
The LUMO and HOMO of Bis-TPE and Bis-TPE with Sc^3+^.

DFT calculations at the B3LYP/6-31+G(d) level revealed that the HOMO–LUMO energy gap of Bis-TPE increased upon Sc^3+^ coordination (Fig. 9). This enlarged gap is in good agreement with the observed fluorescence blue shift (from 559 nm to 520 nm) and the enhanced emission intensity, as a larger energy gap corresponds to higher-energy emission. These results provide strong theoretical support for the experimental observations.

### Application in test paper

**Fig. 10:**
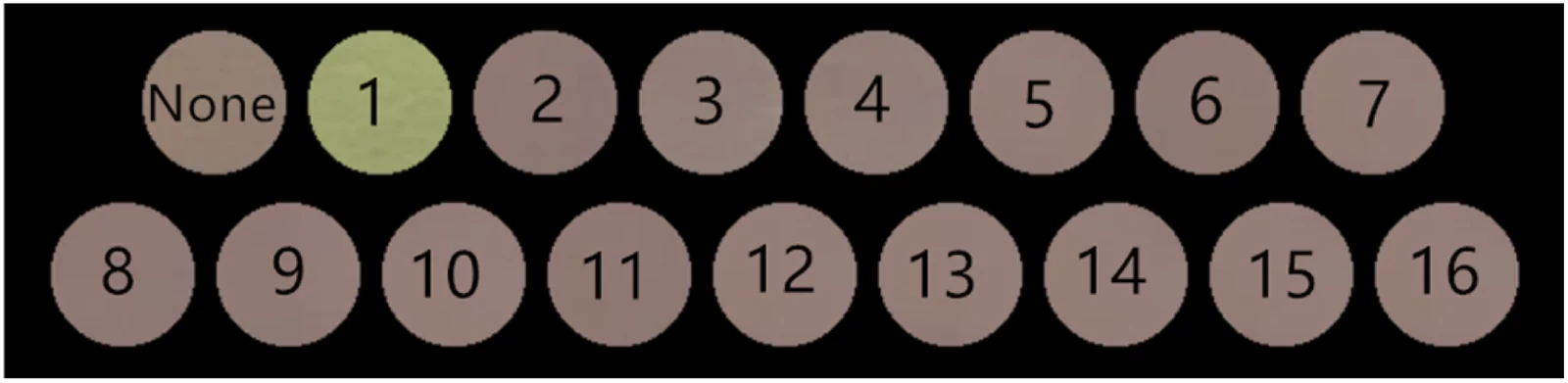
Fluorescence photographs of Bis-TPE with corresponding ion under UV light (365 nm). 1 = Sc^3+^, 2 = Li^+^, 3 = Al^3+^, 4 = Na^+^, 5 = K^+^, 6 = Cd^2+^, 7 = Y^3+^, 8 = Lu^3+^, 9 = Sb^3+^, 10 = Mg^2+^, 11 = Bi^3+^, 12 = Ca^2+^, 13 = La^3+^, 14 = Ce^3+^, 15 = Tl^3+^, 16 = Ag^+^.

In order to verify whether the probe Bis-TPE can be used in real life, a test paper experiment was carried out in this paper. Soak the neutral filter paper in 1 × 10^−5^ mol L^−1^Bis-TPE solution for 5 minutes, dry it naturally, cut it into a round shape, and then add 4 drops of 1 × 10^−4^ mol L^−1^Bis-TPE solution to the filter paper Sc^3+^, Li^+^, Al^3+^, Na^+^, K^+^, Cd^2+^, Y^3+^, Lu^3+^, Sb^3+^, Mg^2+^, Bi^3+^, Ca^2+^, La^3+^, Ce^3+^, Tl^3+^, Ag^+^, the results after drying can be shown in Fig. 10. In Fig. 10, it is found that the filter paper dripped with Sc^3+^ has obvious yellow green color, while the circular filter paper dripped with other ions has no obvious change and is still orange yellow. This indicates that Bis-TPE has good selectivity in species detection.

### Application for sensing Sc^3+^ in water sample

In order to evaluate the application of Bis-TPE for sensing Sc^3+^ in the actual environment, the samples of tap water, river water, and lake water with different amounts of Sc^3+^ were prepared and then examined by fluorescence spectra. The Sc^3+^ contents were calculated by standard working curve and compared with the original concentrations as summarized in Table 1. It can be seen that the Sc^3+^ concentration detected by Bis-TPE in different water samples was close to the original concentrations with the small errors (<4%). These results indicated the excellent application prospect for detecting Sc^3+^ in actual water environments.

**Table 1:** Recovery results of Sc^3+^ in actual water samples

Sample	Found	Added (µM)	Total found (µM)	Recovery (%)	Std dev (%)
Tap water	0	1	0.92	92.0	1.5
	0	2	1.97	98.5	2.1
	0	3	2.93	97.7	3.2
River water	0	1	0.96	96.0	2.1
	0	2	1.92	96.0	3.2
	0	3	2.91	97.0	1.8
Lake water	0	1	0.93	93.0	1.9
	0	2	1.89	94.5	2.7
	0	3	2.92	97.3	3.5

## Conclusions

In summary, we have developed a novel “turn-on” AIE-based fluorescent sensor, Bis-TPE, for the selective detection of Sc^3+^. The probe was synthesized *via* condensation of o-formyl hydroxyltetraphenylethylene with isophthalic dihydrazide in 85% yield. Fluorescence sensing experiments demonstrated that Bis-TPE exhibits high selectivity for Sc^3+^ over other metal ions, including a series of lanthanides, in THF/H_2_O (5 : 95, v/v) mixed medium, with a significant fluorescence enhancement upon Sc^3+^ binding. The detection limit was determined to be 3.57 × 10^−8^ M. A 1 : 1 binding stoichiometry between Bis-TPE and Sc^3+^ was confirmed by fluorescence titration, FT-IR, ^1^H NMR, and mass spectrometry. The practical applicability of the probe was further evaluated using test strips and simulated water samples, demonstrating its potential for environmental monitoring. This work provides a useful molecular design strategy for the development of AIE-based sensors for Sc^3+^ detection in aqueous-organic mixed media.

## Experimental

### General

All chemical reagents were obtained from commercial suppliers and used directly. The other organic solvents and inorganic reagents were pre-treated by standard anhydrous methods before use. TLC analysis was performed on pre-coated glass plates. Column chromatography was carried out by using silica gel (200–300 mesh). NMR spectra were examined in DMSO on a Bruker-ARX 400 instrument at 25 °C. Chemical shifts are reported in ppm with tetramethylsilane (TMS) as internal standard. MS spectra were measured from Bruker mass spectrometer. Compound 1 was synthesized by treating 2-hydroxybenzophenone and benzophenone in Zn, TiCl_4_/THF system according to the published literature.^16^ UV-vis spectra were recorded on varian UV-vis spectrometer. Fluorescence spectra were investigated in a conventional quartz cell (10 × 10 × 45 mm) at 25 °C on a Hitachi F-4500 spectromete. The fluorescence absolute *Φ*_F_ values of solution were collected using an Edinburgh Instruments FLS920 Fluorescence Spectrometer with a 6-inch integrating sphere. All theory calculations were obtained by the density functional theory (DFT) method at the B3LYP/6-31G(d) level by Gaussian 09 program.

## Author contributions

Shengjie Jiang: writing – original draft. Yonglin He: formal analysis. Weimin Yang: data curation.

## Conflicts of interest

There are no conflicts to declare.

## Supplementary Material

RA-016-D6RA06208C-s001

## Data Availability

The work described has not been submitted elsewhere for publication, in whole or in part, and all the authors listed have approved the manuscript that is enclosed. All authors have contributed to the work, have read the manuscript, and have agreed to be listed as authors. The submitted manuscript has not been published elsewhere and nor is it currently under review by another publication. All data needed to evaluate the conclusions in the paper arepresent in the paper. We would like to thank you for your arrangement of the review process. Supplementary information (SI) is available. See DOI: https://doi.org/10.1039/d6ra06208c.
