# Supplementary material for: “Turn-on” fluorescence sensor for Sc3+ based on Schiff-based tetraphenylethylene
Source: RSC Adv. 2026 Aug 24;16(42):50187–92. doi: 10.1039/d6ra06208c (PMC13502254; doi:10.1039/d6ra06208c)
Supplement: Supplementary file 1 [file RA-016-D6RA06208C-s001.pdf]

Supplementary Information (SI) for RSC Advances.
This journal is © The Royal Society of Chemistry 2026

Supporting Information

"Turn-on" fluorescence sensor for Sc^{3+} based on Schiff-based tetraphenylethylene

Shengjie jiang,* [a] Yonglin He, [a] and Weimin Yang [a]

[a] *School of Petroleum and Chemical Engineering, Zhangzhou Institute of Technology, ZhangZhou 363000, P. R. China*

E-mail: jiangshengjie@fjzzit.edu.cn

The synthetic processes of target compounds and the characteristic spectra.

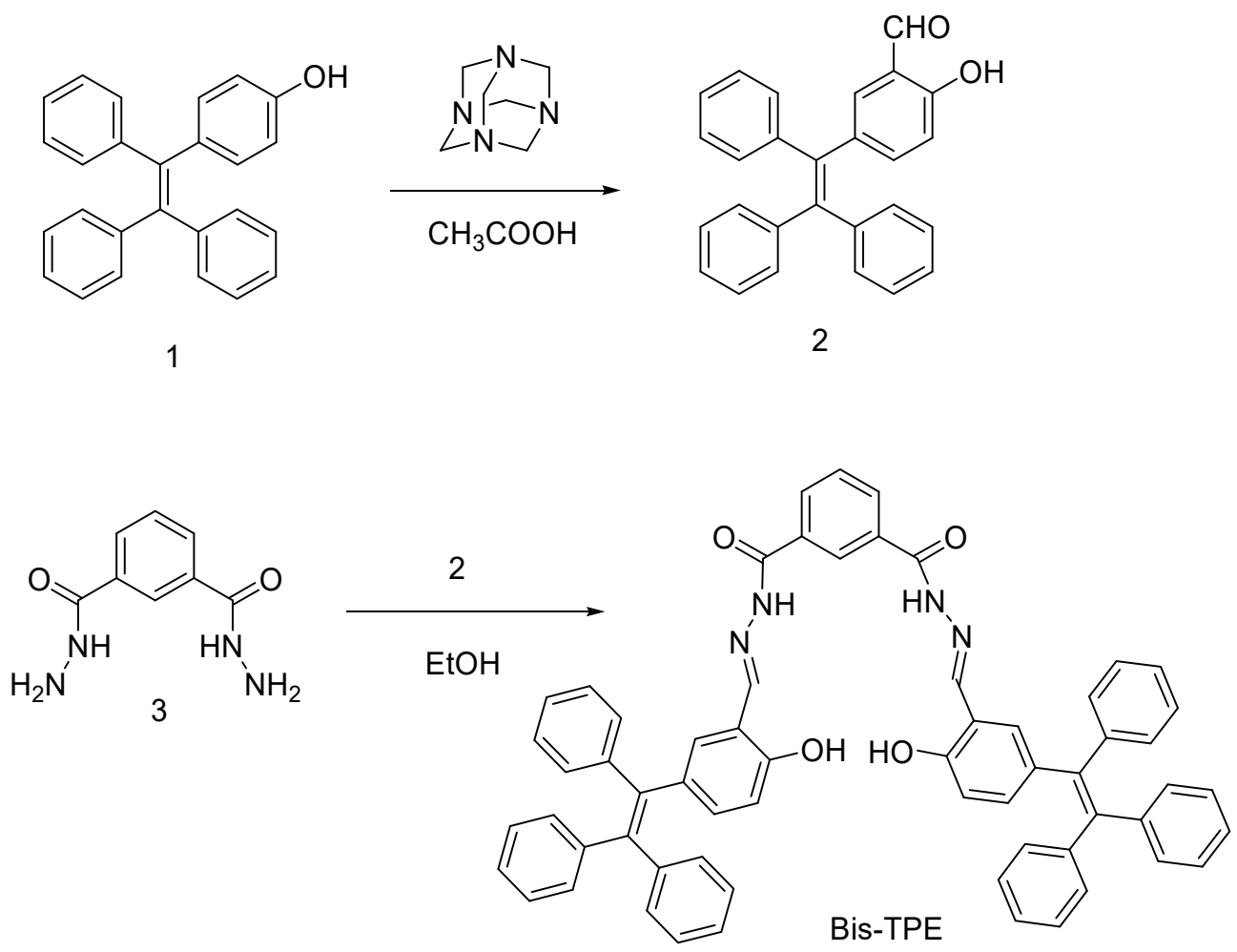

Scheme S1. The synthetic route of title compounds **Bis-TPE**

1. Synthesis procedure of Bis-TPE.

The mixture of compound 2 (0.376 g, 1 mmol) and isophthalic dihydrazide (0.097 g, 0.5 mmol) was added to 20 mL of absolute ethanol, and the mixture was stirred under reflux for 24 h. TLC was used for detection. After the reaction, the solvent was removed under reduced pressure, and the residue was recrystallized from $CHCl_3$/MeOH to obtain a pale yellow powder Bis-TPE (yield 85%).^{1}H NMR (400 MHz, $CDCl_3$) δ 12.14 (s, 2H, NH), 11.10 (s, 2H, OH), 8.47 (d, 2H, CH), 8.11 (d, 4H, ArH), 7.69 (d, 2H, NH), 7.18-6.96(m, 28H, ArH), 6.88 (bs,4H, ArH), 6.70 (d, 4H, J = 8.0 Hz, ArH). ^{13}C NMR (100 MHz, $CDCl_3$), δppm: 162.62, 156.49, 148.27, 143.82, 143.75, 143.53, 140.57, 140.22, 134.88, 134.56, 133.65, 131.44, 131.22, 131.14, 128.35, 128.29, 127.11, 126.97, 118.79, 116.43, 40.57, 40.36, 40.16, 39.95, 39.74, 39.53, 39.32. MALDI-TOF-MS ($C_{62}H_{46}N_4O_4$) Calcd.for m/z = 911.05, found: m/z = 934.071 (M^{+}+Na^{+}).

Anal.calcd for $C_{62}H_{46}N_4O_4$: C 81.74, H 5.09, N 6.15; found C 81.26, H 5.11, N 6.23.

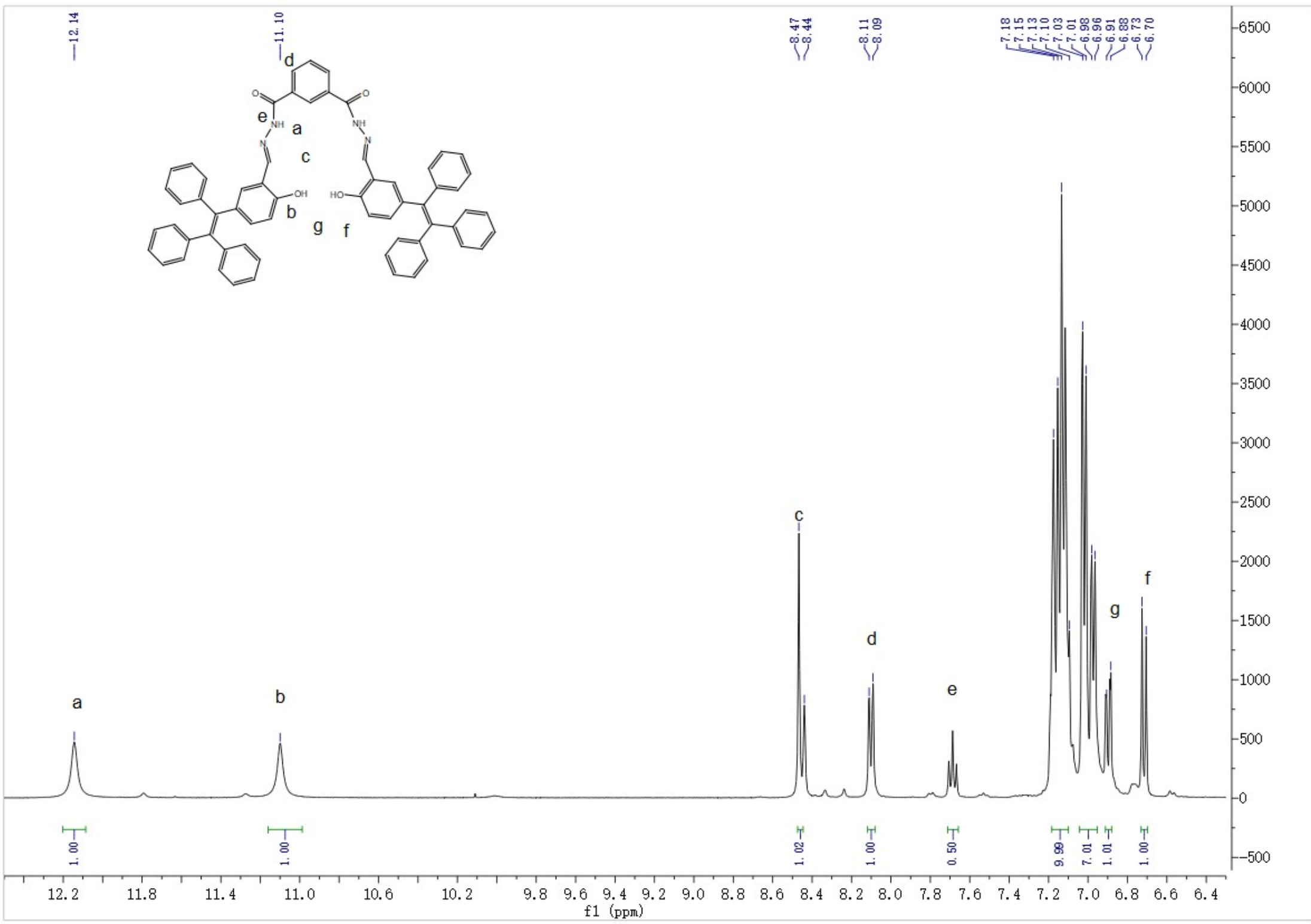

Fig.S1 The ^{1}H NMR spectrum of compound **Bis-TPE**

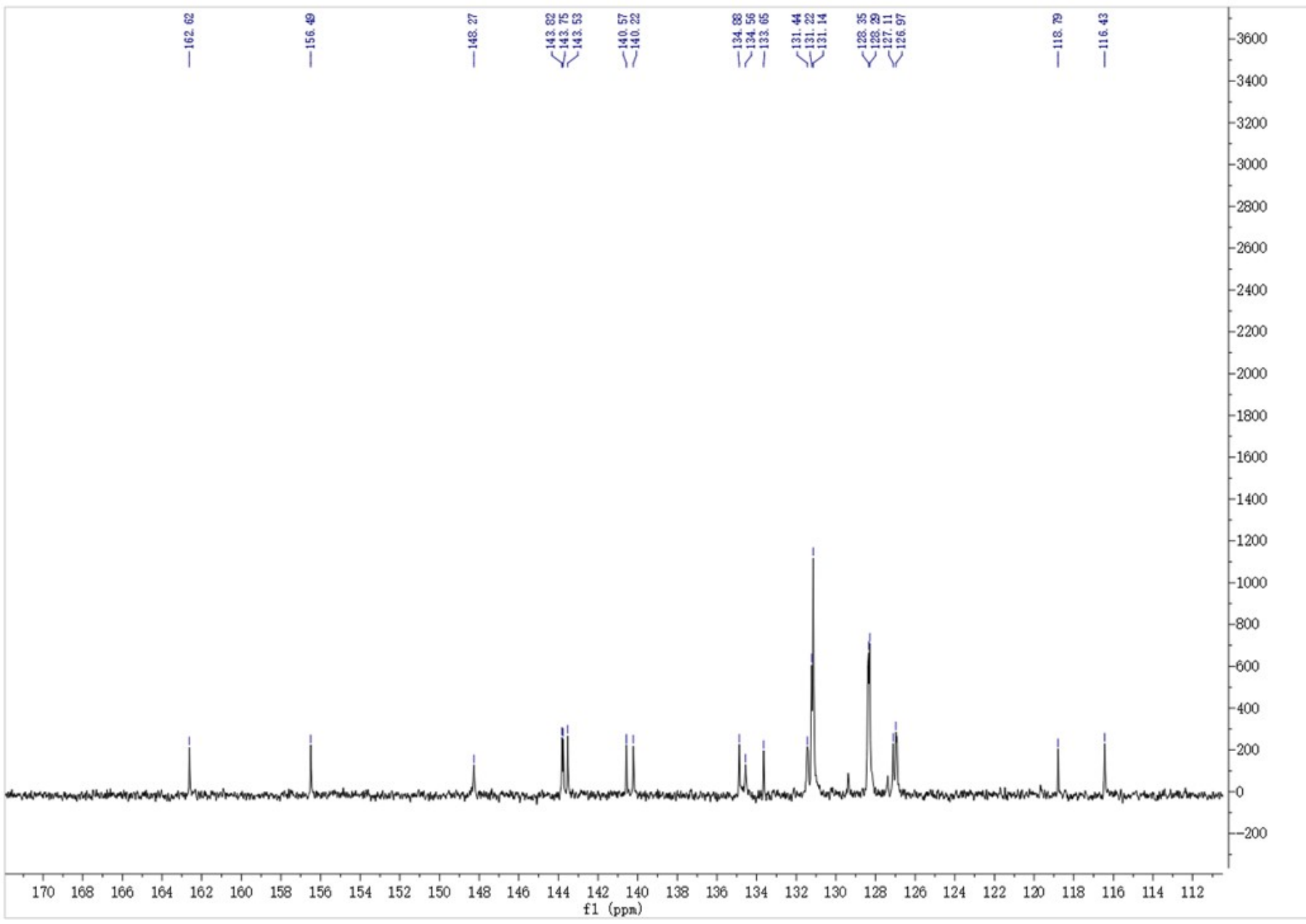

Fig.S2 The ^{13}C NMR spectrum of compound **Bis-TPE**

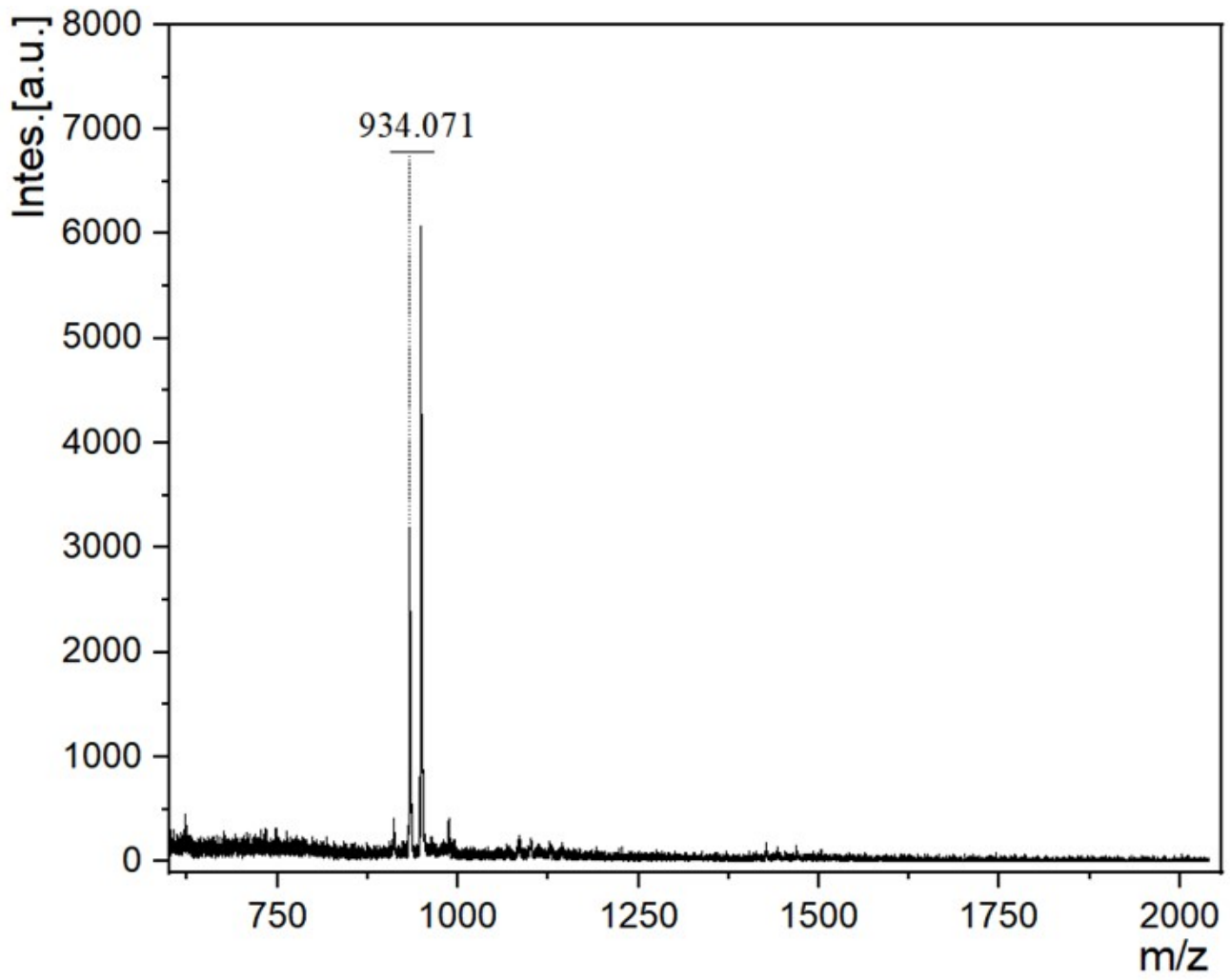

Fig. S3 MALDI-TOF-MS spectrum of compound **Bis-TPE**

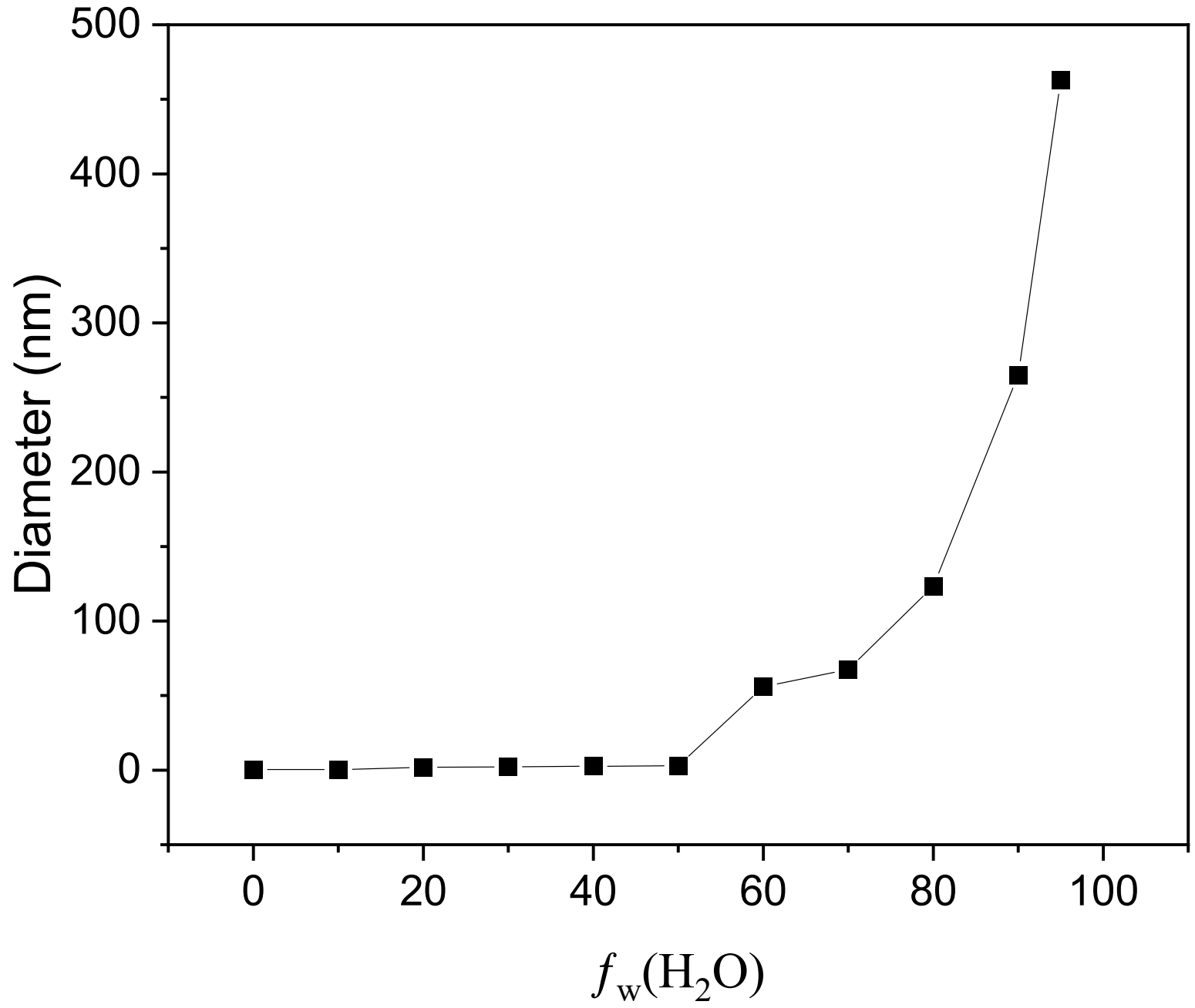

Fig.S4 Hydrodynamic particle size variation curves of luminogens under different water fractions measured by dynamic light scattering (DLS).

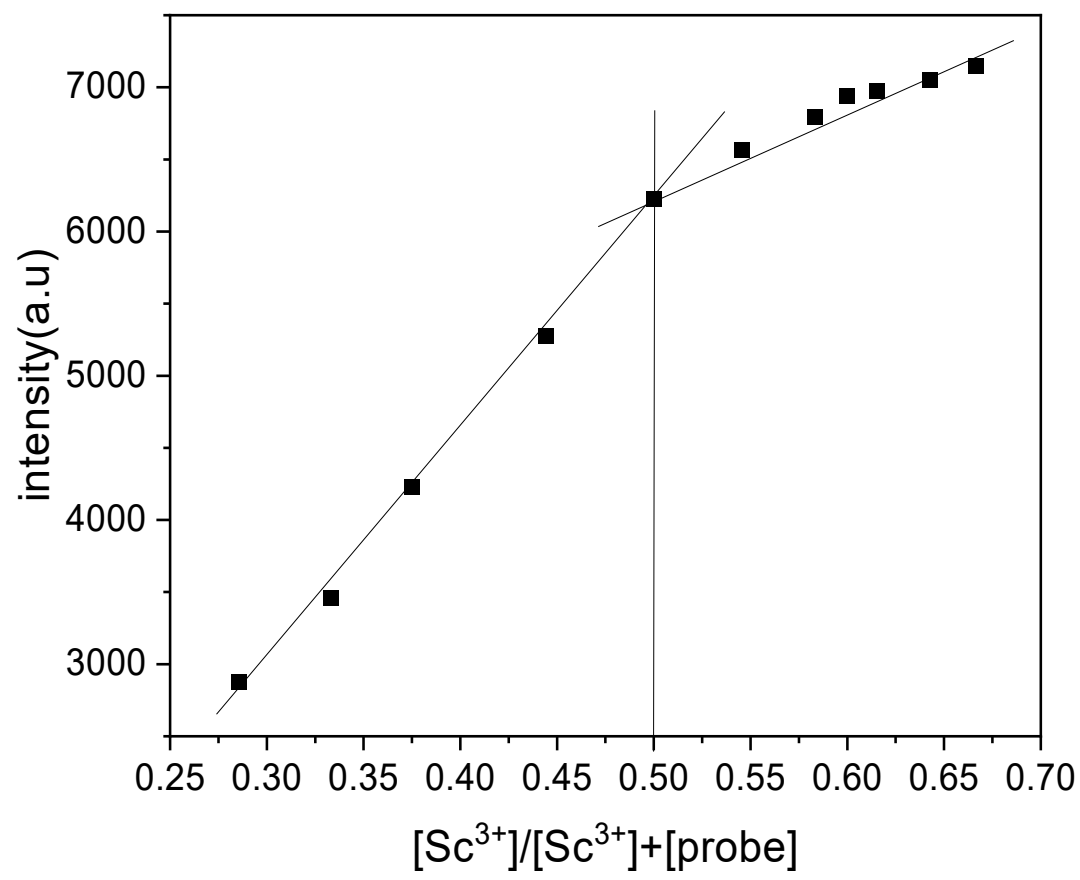

Fig. S5 Fluorescence Jobs' plot (λ_{em} = 520 nm, λ_{ex} = 360 nm) of **Bis-TPE** and Sc^{3+} in THF-H_2O (5:95), the total concentration of [**Bis-TPE**] + [Sc^{3+}] = 20 μM.

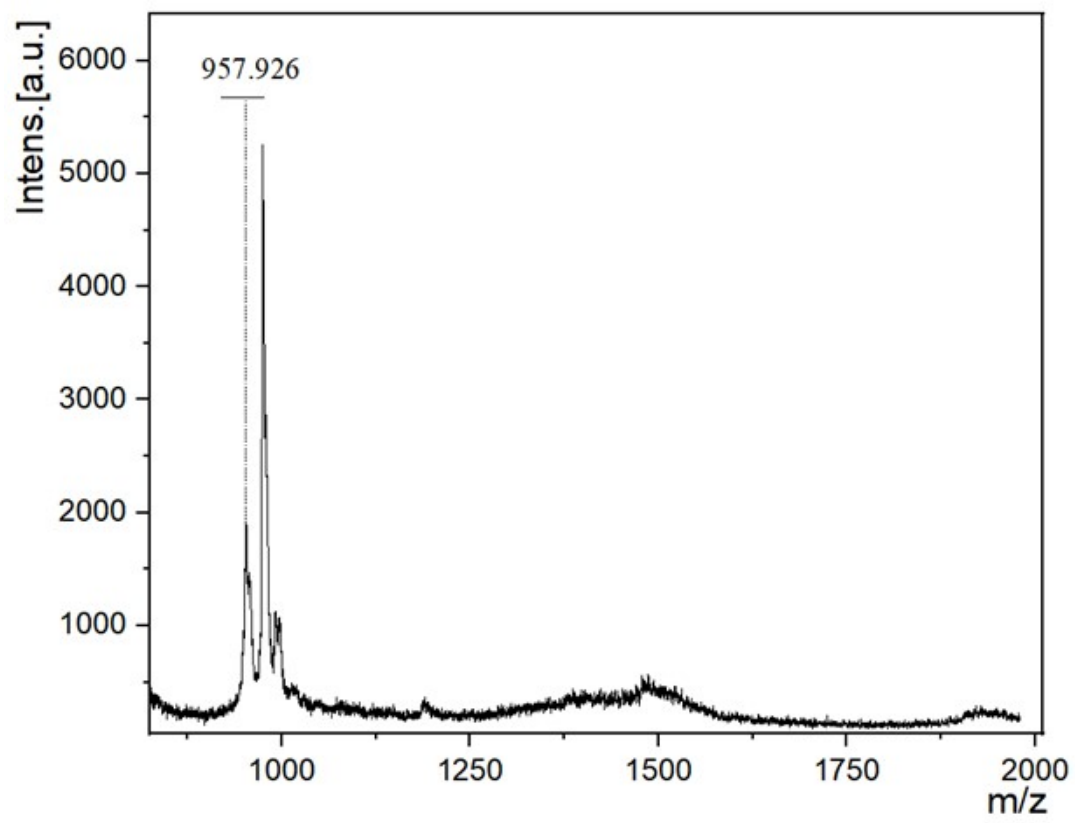

Fig. S6 The MALDI-TOF-MS spectrum of **Bis-TPE** with Sc^{3+} (1:4)

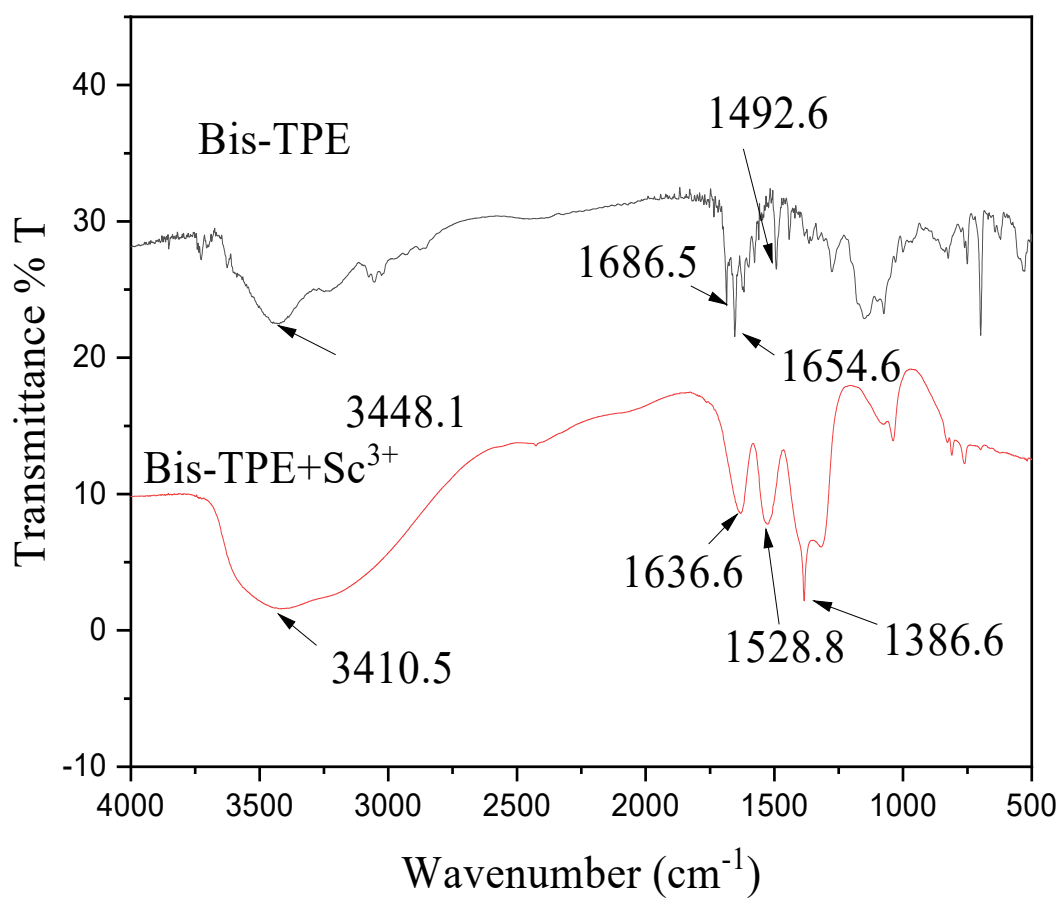

Fig. S7 The FT-IR spectra of **Bis-TPE** and **Bis-TPE** with Sc^{3+} (1:1).
